# Humanistic and Economic Burden of Conversion Therapy Among LGBTQ Youths in the United States

**DOI:** 10.1001/jamapediatrics.2022.0042

**Published:** 2022-03-07

**Authors:** Anna Forsythe, Casey Pick, Gabriel Tremblay, Shreena Malaviya, Amy Green, Karen Sandman

**Affiliations:** 1 Value and Access, Cytel Inc, Waltham, Massachusetts; 2 The Trevor Project, West Hollywood, California; 3 Health Economics, Cytel Inc, Montreal, Quebec, Canada; 4 Health Economics, Cytel Inc, Waltham, Massachusetts

## Abstract

**Importance:**

Sexual orientation and gender identity change efforts (SOGICE), also called *conversion therapy*, is a discredited practice attempting to convert lesbian, gay, bisexual, transgender, queer, or questioning (LGBTQ) individuals to be heterosexual and/or cisgender.

**Objectives:**

To identify and synthesize evidence on the humanistic and economic consequences of SOGICE among LGBTQ youths in the US.

**Design, Setting, and Participants:**

This study, conducted from December 1, 2020, to February 15, 2021, included a systematic literature review and economic evaluation. The literature review analyzed published evidence on SOGICE among LGBTQ individuals of any age. The economic model evaluated the use of SOGICE vs no intervention, affirmative therapy vs no intervention, and affirmative therapy vs SOGICE to estimate the costs and adverse outcomes for each scenario and to assess the overall US economic burden of SOGICE. Published literature and public sources were used to estimate the number of LGBTQ youths exposed to SOGICE, the types of therapy received, and the associated adverse events (anxiety, severe psychological distress, depression, alcohol or substance abuse, suicide attempts, and fatalities).

**Exposures:**

SOGICE (licensed or religion-based practitioners) or affirmative therapy (licensed practitioners).

**Main Outcomes and Measures:**

Total incremental costs and quality-adjusted life-years (QALYs) vs no intervention and total economic burden of SOGICE.

**Results:**

Among 28 published studies, which included 190 695 LGBTQ individuals, 12% (range, 7%-23%) of youths experienced SOGICE, initiated at a mean age of 25 years (range, 5-58 years), with a mean (SD) duration of 26 (29) months. At least 2 types of SOGICE were administered to 43% of recipients. Relative to LGBTQ individuals who did not undergo SOGICE, recipients experienced serious psychological distress (47% vs 34%), depression (65% vs 27%), substance abuse (67% vs 50%), and attempted suicide (58% vs 39%). In the economic analysis, over a lifetime horizon with a 3% annual discount rate, the base-case model estimated additional $97 985 lifetime costs per individual, with SOGICE associated with 1.61 QALYs lost vs no intervention; affirmative therapy yielded cost savings of $40 329 with 0.93 QALYs gained vs no intervention. With an estimated 508 892 youths at risk for SOGICE in 2021, the total annual cost of SOGICE is estimated at $650.16 million (2021 US dollars), with associated harms totaling an economic burden of $9.23 billion.

**Conclusions and Relevance:**

This economic evaluation study suggests that there is a high economic burden and high societal costs associated with SOGICE and identifies additional research questions regarding the roles of private and public funding in supporting this harmful practice.

## Key Points

**Question:**

What is the total economic cost of sexual orientation and gender identity change efforts (SOGICE), also called *conversion therapy*, including adverse consequences, among lesbian, gay, bisexual, transgender, queer, or questioning (LGBTQ) youths in the US?

**Findings:**

This systematic literature review and economic evaluation found that the total annual cost of SOGICE among 4 554 300 LGBTQ youths in the US is estimated at $650.16 million, with associated harms, such as substance abuse and suicide attempts, totaling an estimated total economic burden of $9.23 billion.

**Meaning:**

This study suggests that, in addition to being detrimental from a clinical and humanistic standpoint, SOGICE and their harmful effects among LGBTQ youths in the US are estimated to cost billions of dollars each year.

## Teaser

This economic evaluation study with a systematic literature review identifies and synthesizes evidence on the humanistic and economic consequences of sexual orientation and gender identity change efforts among LGBTQ (lesbian, gay, bisexual, transgender, queer, or questioning) youths in the US.

## Introduction

Sexual orientation and gender identity change efforts (SOGICE) (also called *conversion therapy*) are dangerous, discredited practices rooted in false beliefs that being lesbian, gay, bisexual, transgender, queer, or questioning (LGBTQ) is pathologic.^1^ Based on evidence that SOGICE is ineffective and detrimental,^2^ the American Academy of Pediatrics, the American Academy of Child and Adolescent Psychiatry, and other medical, mental health, and human rights organizations formally oppose it.^3,4,5,6,7,8,9,10^

Minority stress theory contends that disproportionate rates of health issues among LGBTQ individuals stem from chronic stress and mental health detriments caused by increased exposure to social bigotry and rejection.^11^ SOGICE reinforces societal prejudices and stigmas through promoting sexual and gender identity rejection.^11^ For already vulnerable youths, it may exacerbate distress or incite guilt, shame, and self-hatred and is associated with devastating mental and physical health consequences, including new or increased depression, anxiety, self-harm, suicidal ideation, nightmares, gastrointestinal distress, sexual dysfunction, relationship problems, and isolation.^3,11,12^

As of August 2021, 25 states, the District of Columbia, and Puerto Rico have instituted bans on or executive orders protecting minors from SOGICE^1^; unlicensed individuals such as religious practitioners are not regulated. Per a 2019 study, 698 000 LGBTQ adults in the US have undergone SOGICE; approximately half underwent it as minors.^13^ Many of these individuals require therapy and support to address the harms of SOGICE.

Limited research has synthesized the overall clinical, humanistic (ie, quality-of-life), and economic burden of SOGICE. To inform legal and health care policy makers, we conducted a systematic literature review (SLR) and economic evaluation to quantify the consequences of SOGICE, focusing on adolescents and young adults, who are especially vulnerable and common targets.^13^

## Methods

This economic evaluation study, conducted from December 1, 2020, to February 15, 2021, included an SLR, which comprised LGTBQ individuals of any age. The SLR was conducted to compile a broad evidence base regarding SOGICE and its effects and to support the economic model, focusing on key research questions:

- How many individuals, particularly adolescents and young adults, in the US have undergone SOGICE?
- What are the types and duration of therapy?
- What are the humanistic and economic harms of SOGICE?
- What are the health care resources and costs associated with SOGICE?

Detailed methods are outlined in the eMethods, eTable 1, and eFigure 1 in the Supplement. This economic evaluation used data only from previously published literature; such data were deidentified, publicly available, and protected by prior consent. This study followed the Consolidated Health Economic Evaluation Reporting Standards (CHEERS) reporting guideline, the Preferred Reporting Items for Systematic Reviews and Meta-analyses (PRISMA) reporting guideline, and the National Institute for Health and Care Excellence guideline.^14,15,16,17^

A decision-tree model was developed in Excel, version 2018 for Microsoft 365 (Microsoft Corp) to assess the costs and consequences of SOGICE vs no intervention, affirmative therapy vs no intervention, and affirmative therapy vs SOGICE, supplemented with an economic evaluation to assess the overall US economic burden of SOGICE. In this analysis, we defined *affirmative therapy* as psychotherapy validating the positive expression of sexual and gender identities and recognizing the association of macrolevel forces, such as heterosexism and homophobia, with well-being.^18^

**Figure 1.**
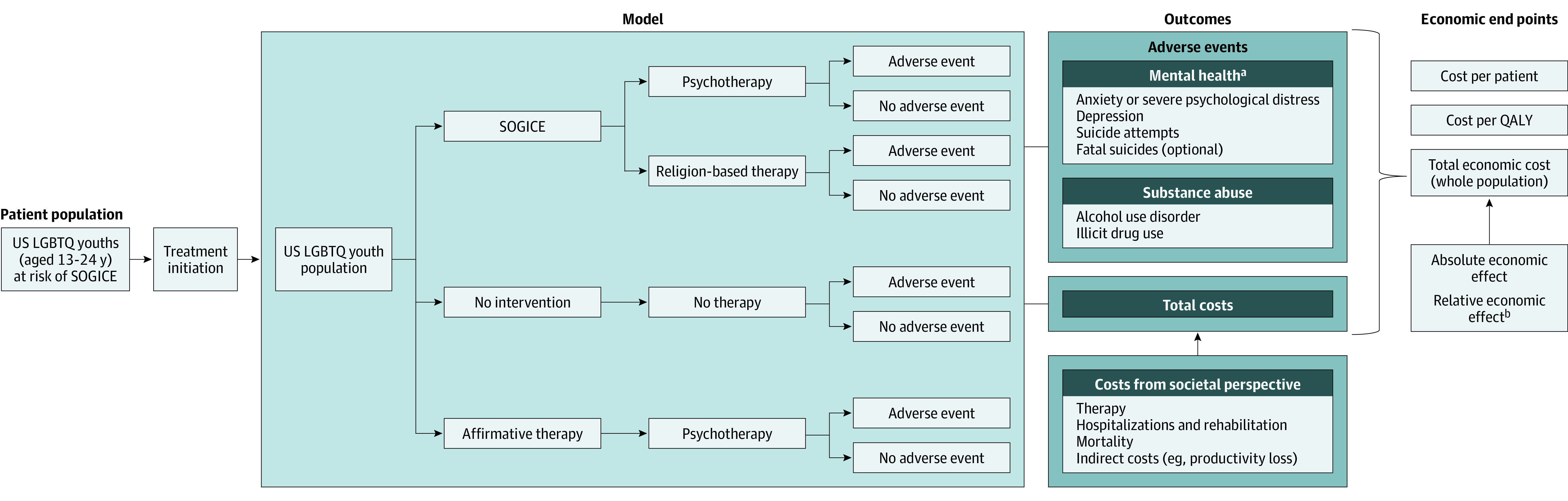
Model Framework LGBTQ indicates lesbian, gay, bisexual, transgender, queer, or questioning; QALY, quality-adjusted life-year; and SOGICE, sexual orientation and gender identity change efforts. ^a^Because the adverse events are not mutually exclusive, the decision tree evaluates these outcomes separately. The adverse event can be anxiety or depression or suicide attempts with or without fatal suicide or alcohol use disorder or illicit drug use. ^b^The absolute economic effect is the difference in total economic costs between the interventions. The relative economic effect is the percentage change in economic costs for an intervention when compared with another.

The decision-tree structure accounted for various SOGICE modalities (psychotherapy and religion-based therapy), which incur different costs owing to factors such as type of practitioner and duration of therapy. The model evaluated the probability of therapy outcomes (adverse events) in the at-risk population, such as anxiety, severe psychological distress, depression, alcohol or substance abuse, suicide attempts, and fatal suicide attempts (Figure 1). For simplicity, the decision tree considered the costs and consequences of adverse events separately (ie, if an individual experienced anxiety and alcohol use disorder, then these were considered as discrete events and not as a combined adverse event).

The analysis was conducted from the US societal perspective; both direct costs (costs of treatment, health outcomes, and mortality) and indirect costs (ie, costs such as productivity loss that are not directly incurred by therapy and its outcomes) were analyzed. The base-case analysis considered a lifetime horizon, with costs and effectiveness discounted at 3% annually.

### Assumptions

The model made several assumptions because of the limited availability of data. First, it assumed the same likelihood of adverse outcomes regardless of SOGICE method. It also assumed that individuals of all sexual orientations, gender identities, and ages experience the same likelihood of outcomes, costs, and quality-of-life utility values. The likelihood of outcomes for affirmative therapy was calculated using the relative risk reduction and relative risk estimates applied to the no-intervention likelihoods of each outcome. Furthermore, it was assumed that the suicidality score used in this estimation is indicative of the likelihood of a suicide attempt. The model also considered the costs associated with suicide attempts that require acute medical care. Equivalent costs were assumed for rehabilitation for alcohol use disorder and substance abuse. The model assumes that people receive 1 type of therapy, although, in principle, an individual could receive multiple therapy modalities. The assumed cost of psychotherapy (60-minute individual sessions with a licensed mental health professional; *Current Procedural Terminology* code 90837) was $142. The model also assumed that individuals experience the health-state utility values and costs related to therapy and adverse events for up to 3 years. Last, given the dearth of LGBTQ-specific suicide mortality data, the likelihood of fatal suicide on index attempt was assumed to be equivalent across all interventions and no different than the likelihood observed in the general population.

### Model Inputs

Model inputs were based on the most current and robust evidence sources identified in the SLR, to ensure that the inputs reflected the current experiences of LGBTQ adolescents and young adults in the US.

#### Population

The target population was LGBTQ adolescents and young adults aged 13 to 17 years in the states where SOGICE directed at minors is legal (n = 1 157 000),^19^ LGBTQ adolescents and young adults aged 13 to 17 years in states where SOGICE directed at minors is illegal but religious efforts are permitted (n = 835 000),^19^ and LGBTQ adolescents and young adults aged 18 to 24 years nationally (n = 3 313 800).^20,21^ Approximately 10% of the target population is considered at risk for receiving SOGICE.^22^ For minors in states in which SOGICE is illegal, the at-risk population for SOGICE was assumed to be those who would undergo religious therapy. An estimated 43% of the target population aged 16 to 24 years is employed and will experience indirect costs from adverse events.^23^

#### Type of Therapy

We analyzed the outcomes of 2 key methods: therapy provided by licensed health professionals (26%) and religion-based SOGICE (74%).^13^ The base case considered these modalities to be mutually exclusive.

#### Adverse Outcomes

The probabilities of adverse events experienced by LGBTQ youths undergoing SOGICE as compared with no intervention and with affirmative therapy were estimated using data obtained from the SLR (Table 1).^24,25,26,27,28,29,30^ The relative effectiveness of affirmative therapy was indirectly calculated using estimates of relative risk reduction and relative risks (eTable 2 in the Supplement) for each adverse health outcome.

**Table 1.** Likelihood of Health Outcomes by Intervention

Health outcome	LGBTQ youths, %		
	No intervention	SOGICE	Affirmative therapy
Anxiety or severe psychological distress	34^24^	47^24^	20^25^
Depression	27^26^	65^26^	14^25^
Alcohol use disorder	42^26^	41^26^	30^25^
Illicit drug use	50^26^	67^26^	26^27,28^
Index suicidal attempt	22^26^	63^26^	3^29^
Fatal suicide on index attempt	0.9^28,29,30^	2.5^28,29,30^	0.1^28,29,30^

Abbreviations: LGBTQ, lesbian, gay, bisexual, transgender, queer, or questioning; SOGICE, sexual orientation and gender identity change efforts.

#### Utilities

Utility inputs for different health states in the model are summarized in eTable 3 in the Supplement. The SLR did not identify published evidence on quality of life or utility values in LGBTQ youths. Additional searches were conducted to obtain utility inputs associated with health outcomes, which were assumed to be the same regardless of the intervention.^31,32,33^ The model assumed that the health-state utility values were experienced for 3 years and then returned to baseline. With the use of Centers for Disease Control and Prevention life tables, natural mortality was used to generate lifetime survival for all health states except fatal suicide or death.^34^ Lifetime survival was used to estimate quality-adjusted life-years (QALYs) after 3 years over a lifetime using the baseline value of 0.865, which is lower than that of the general population, in accordance with the minority stress theory.

#### Costs

The model considered the costs associated with interventions and adverse events (eTable 4 in the Supplement).^33,35,36,37,38,39,40,41,42,43,44,45,46,47^ All costs were estimated for 3 years, after which the population was assumed to not experience further intervention or adverse events and hence incur no further costs. Inflation rates were calculated using the medical care index of the US Consumer Price Index.^48^

The model considered the costs of 2 types of SOGICE (religion-based or licensed practitioners) and the costs of affirmative therapy (administered as psychotherapy and assumed to have the same costs as SOGICE administered by licensed practitioners). The cost per session and the duration of therapy varied by therapy type. The mean (SD) number of sessions was assumed to be 118 (135), with a mean (SD) duration of 26 (29) months.^49^

The direct medical costs and the indirect costs (such as mortality and productivity loss) were obtained and estimated for each health outcome from public data sources and published literature; these costs were not specific to the LGBTQ population but were based on the general population (eTable 5 in the Supplement).^35^

### Model Outputs

#### Base-Case Analysis

Total costs were calculated separately for SOGICE, no therapy, and affirmative therapy as the sum of individual cost inputs. Quality-adjusted life-years lost were calculated as mean values per person over the modeled time horizon. The total economic burden of SOGICE in the US, based on 30 states where the practice is legal among minors (13-17 years of age) and states where SOGICE directed at minors is illegal but religious efforts are permitted, and nationwide for young adults (18-24 years of age), was estimated based on these quantitative analyses.

#### Scenario Analysis

The following scenarios were compared for overall association with the final results:

- Varied use of SOGICE by type of therapy was observed by Blosnich et al,^11^ where SOGICE was provided by health care professionals (31%) and/or religious leaders (81%). Therapy with health care professionals was assumed to cost the same as psychotherapy.
- Indirect costs (productivity loss and mortality costs) were excluded owing to uncertainty associated with estimates.
- Utilities for adverse events were reweighted based on a baseline utility of 0.865 instead of 1.
- Lifetime likelihood of fatality due to suicide reattempt (2%) within 1 year of the episode period (3-year period during which individuals experience health-state utility and costs associated with therapy and adverse events in the model) was included.^30^
- Fatal suicide attempts were excluded from the model.

## Results

### Systematic Literature Review

The SLR provided a broad view of the published evidence regarding SOGICE; selected recent and robust sources from the SLR were used as inputs in the economic model. The 28 publications^11,12,13,22,24,25,26,27,49,50,51,52,53,54,55,56,57,58,59,60,61,62,63,64,65,66,67,68,69,70,71,72^ identified comprised 190 695 LGBTQ individuals; among these publications, overall, 12% (range, 7%-23%) of youths experienced SOGICE, including individual or group psychotherapy (31%-100%), inpatient SOGICE (7%), and SOGICE administered by religious leaders (18%-81%). SOGICE was initiated at a mean age of 25 years (range, 5-58 years), with a mean (SD) of 118 (135) sessions per individual and a mean (SD) duration of treatment of 26 (29) months. The literature was not sufficient to determine the overall proportion of individuals starting SOGICE as minors vs adults. At least 2 types of SOGICE were administered to 43% of recipients, with 15% undergoing more than 3 modalities.

Relative to LGBTQ individuals who did not undergo SOGICE, those who did undergo SOGICE experienced severe consequences, including serious psychological distress (47% vs 34%), depression (65% vs 27%), problematic substance use (67% vs 50%), attempted suicide (58% vs 39%; odds ratio, 2.27 [95% CI, 1.60-3.24; *P* < .001]), and attempted suicide causing moderate or severe injury (67% higher odds; odds ratio, 1.67 [95% CI, 0.76-3.64]).

### Economic Evaluation

**Figure 2.**
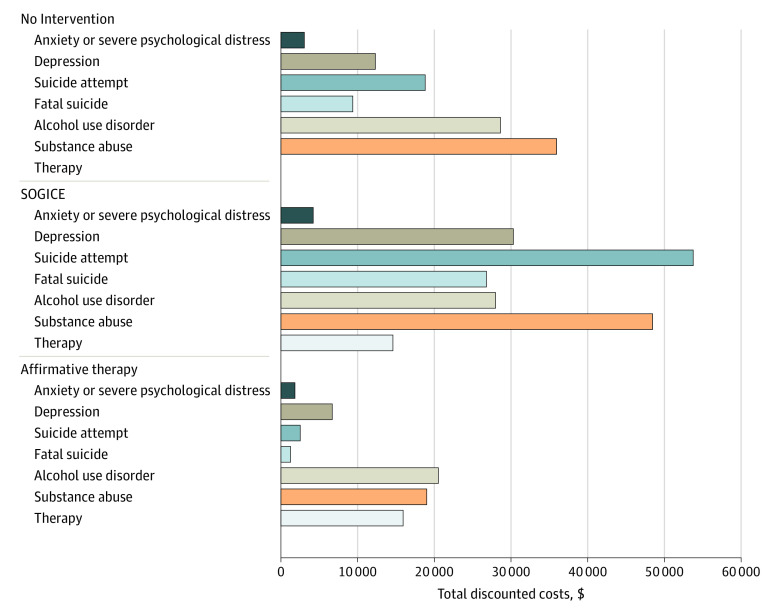
Total Discounted Costs Per At-Risk Individual by Intervention Type SOGICE indicates sexual orientation and gender identity change efforts.

Over a lifetime horizon, LGBTQ youths who received SOGICE were expected to incur a total discounted cost of $206 159 vs $108 174 for no therapy and $67 844 for affirmative therapy per individual at risk (Figure 2). The direct medical discounted cost incurred for SOGICE was $148 098 vs $85 292 for no therapy and $62 056 for affirmative therapy. The direct cost of affirmative therapy was higher ($15 936) compared with SOGICE ($14 619) because affirmative therapy was provided only by licensed medical professionals, whereas SOGICE was provided by licensed practitioners as well as unlicensed religion-based professionals. Although SOGICE administration costs were lower than for affirmative therapy, costs associated with adverse health outcomes were $139 632 higher with SOGICE. The primary factors associated with the costs for SOGICE were outcomes including suicide attempts, fatal suicide attempts, depression, and substance abuse. Overall discounted QALYs were 23.30 for SOGICE, 24.91 for no therapy, and 25.84 for affirmative therapy (Table 2).

**Table 2.** Cost-Utility Analysis Comparing No Intervention With SOGICE and Affirmative Therapy

Intervention	Discounted		Incremental^a^^,^^b^		Interpretation of ICURs
	Costs, $	QALYs	Costs, $	QALYs	
No intervention	108 174	24.91	NA	NA	NA
SOGICE	206 159	23.30	97 985	(1.61)	SOGICE dominated
Affirmative therapy	67 844	25.84	(40 329)	0.93	Affirmative therapy dominated

Abbreviations: ICURs, incremental cost-utility ratios; NA, not applicable; QALYs, quality-adjusted life-years; SOGICE, sexual orientation and gender identity change efforts.

^a^ Values in parentheses are negative.

^b^ Incremental values are based on unrounded, exact values, not the rounded costs presented in the table.

The economic model estimated that SOGICE was associated with 1.61 QALYs lost at an additional cost of $97 985, whereas affirmative therapy was associated with an increase of 0.93 QALYs with a $40 329 cost decrease vs no therapy (Table 2). The model estimated that affirmative therapy would be associated with $138 315 in decreased costs for 2.53 QALYs gained vs SOGICE. Relative to no intervention, SOGICE leads to higher costs and worse outcomes, whereas affirmative therapy leads to lower costs and better outcomes compared with both SOGICE and no therapy. The results of the scenario analysis agreed with the results of the base-case analysis (eTable 6 in the Supplement).

With an estimated 508 892 LGBTQ youths at risk to receive SOGICE in 2021 based on reported rates of therapy (eFigure 2 in the Supplement), total SOGICE costs were estimated at $650.16 million, with harms associated with an estimated economic burden of $8.58 billion, for a total burden of $9.23 billion (Table 3). Although affirmative therapy incurred costs of $709 million vs no therapy, we estimated total savings of $1.81 billion for affirmative therapy in the same population.

**Table 3.** Total Economic Burden by Intervention and Factors Associated With Cost^a^

Intervention	No therapy	SOGICE	Affirmative therapy	SOGICE vs no therapy^b^	Affirmative therapy vs no therapy^b^	Affirmative therapy vs SOGICE^b^
Therapy costs, $	0.00	0.65	0.71	0.65	0.71	0.06
Total costs by health outcomes, $						
Anxiety or severe psychological distress	0.14	0.19	0.08	0.05	(0.06)	(0.11)
Depression	0.55	1.36	0.30	0.81	(0.25)	(1.06)
Suicide attempt	0.85	2.42	0.11	1.57	(0.73)	(2.30)
Fatal suicide	0.41	1.17	0.06	0.76	(0.35)	(1.12)
Alcohol use disorder	1.29	1.26	0.92	(0.03)	(0.36)	(0.34)
Substance abuse	1.62	2.18	0.86	0.56	(0.76)	(1.32)
Total costs, $	4.85	9.23	3.04	4.38	(1.81)	(6.19)

Abbreviation: SOGICE, sexual orientation and gender identity change efforts.

^a^ All costs in billions of 2021 US dollars. Values in parentheses are negative (cost savings).

^b^ Incremental costs are calculated from unrounded, exact costs, not the rounded costs presented in the table.

## Discussion

Despite the increase in public support for LGBTQ individuals in the US,^69^ current published literature reveals that SOGICE reinforces societal prejudices and stigmas through promoting sexual and gender identity rejection.^11^ Approximately 10% of LGBTQ individuals undergo SOGICE in the form of individual or group psychotherapy, inpatient treatment, or administration by religious leaders; many individuals undergo multiple modalities, typically as youths.^22^ SOGICE recipients experience increased rates of psychological distress, depression, substance abuse, and suicidality.^11,12,24^ Although these clinical and humanistic consequences are severe, no published studies have formally evaluated the economic costs of this unnecessary, harmful practice.

To our knowledge, our economic evaluation is the first to assess the association of SOGICE with socioeconomic outcomes. Its findings underscore the costs of inflicting harm on a vulnerable young population. In addition to the resources wasted on SOGICE, the downstream consequences are associated with lifetime excess costs of $83 366 per individual at risk, primarily associated with suicidality, anxiety, severe psychological distress, depression, and substance abuse. From a population perspective, this translated to total costs of $650 million for SOGICE in 2021, with harms associated with an estimated economic burden of $9.23 billion.

The base-case analysis compared SOGICE with no intervention, but affirmative therapy is an economically feasible alternative that may benefit LGBTQ youths by reducing rates of adverse outcomes. The lifetime excess costs per individual decreased by $138 315 for affirmative therapy vs SOGICE. Overall, the potential US savings with affirmative therapy are estimated at $1.81 billion (vs no intervention) and nearly $6.19 billion (vs SOGICE).

This study’s design has several distinct features that allow us to provide robust estimates to inform policy. The key model inputs originated from an SLR, which identified multiple large-scale studies regarding the use and outcomes of SOGICE. The model structure incorporated various methods of SOGICE and a range of literature-reported outcomes. Last, overall estimates of the economic burden of conversion therapy reflect the real-world treatment landscape and are adjusted based on the legality of minor-directed SOGICE in different states at the time of analysis.

### Limitations

This study has some limitations. Given the evolving societal understanding of gender and sexual orientation, this study focused on recent evidence to ensure that the economic analysis reflected current practices and experiences of LGBTQ adolescents and young adults in the US. A key limitation is that, owing to limited data, the model makes several assumptions, including that the risk of adverse outcomes was the same across different sexual orientations and gender identities and for various SOGICE modalities. The likelihood of adverse outcomes was obtained from the published literature based on participant self-reporting, which is associated with bias in the estimates and increases the uncertainty for these values. The estimation of the likelihood of these events for affirmative therapy using a comparative approach introduces additional uncertainty. The literature used to estimate the likelihood of adverse outcomes was selected to reflect the target population of this model (adolescents and young adults aged 13-24 years), but individual publications may focus on teens, young adults, or broader age ranges, adding to the uncertainty. The inclusion of non–LGBTQ-specific health utilities may be associated with an underestimation of the association of these interventions with quality-of-life measures among LGBTQ individuals. There is uncertainty surrounding the costs associated with adverse events over a lifetime horizon because they were obtained from the current published and available evidence, which generally was not specific to the LGBTQ population. Because the estimation of indirect costs was not standardized among publications, using the human capital approach to estimate productivity loss (eg, suicide attempt and fatal suicide attempt) may be associated with further uncertainty and an overestimation of costs.

Although there are various uncertainties associated with this economic model, in general, it took a conservative approach and is likely to underestimate the true economic effect of SOGICE. The model accounts for costs and outcomes associated with adverse events for 3 years after SOGICE, even though harms and their costs may persist much longer, leading to higher costs. Moreover, there may be additional consequences of SOGICE not included in the analysis (eg, the development of eating disorders or posttraumatic stress disorder) as well as medical consequences of some techniques (such as inappropriate use of medications or electroconvulsive therapy). Owing to the limitations and challenges in collecting suicide data, there is also uncertainty associated with the proportion of suicide attempts that may lead to fatalities among the LGBTQ population; the base-case model does not consider the rate and fatality of suicide reattempts.^28,29^ Last, as an economic analysis, this model necessarily examines SOGICE with a focus on direct and indirect monetary costs and cost offsets. Beyond the economic consequences, as noted by multiple international organizations, individuals subjected to this practice experience serious detrimental effects.^3,4,5,6,7,8,9,10^ Limitations in the evidence base represent opportunities for additional research to better understand this diverse, underserved community that is often actively harmed by clinicians, mental health professionals, and unlicensed practitioners.

Although it is possible that there may be selection bias, in that youths who undergo SOGICE are at elevated risk for adverse outcomes owing to a greater level of distress with their gender and/or sexual identity that may lead them to seek conversion therapy, such an argument assumes that they freely seek SOGICE. In a 2020 survey, 58% of LGBTQ youths reported that someone, typically parents, friends, relatives, or religious leaders, attempted to convince them to change their sexual orientation or gender identity.^22^ With such prevalent pressure to change orientation or identity, it is unlikely that LGBTQ individuals who undergo SOGICE differ from their peers except for the extent of the pressure or coercion they receive.

## Conclusions

There are already multiple, unambiguous statements from professional societies and human rights groups on the imperative to stop SOGICE because of its discriminatory nature and profoundly harmful effects. This current analysis adds an economic dimension to the discussion, demonstrating a difference in economic consequences between SOGICE, no intervention, and affirmative therapy. It is incumbent on policy makers to act to protect youths from—and stop all funding for—this unacceptable practice. Likewise, increasing access to affirmative therapy may promote health by empowering LGBTQ youths with skills and strategies to counteract minority stress.^25^

## Notes

Supplement. eMethods. eTable 1. PICOS Criteria for the SLR eTable 2. Estimates of the Relative Risk of Adverse Health Outcomes: Affirmative Therapy vs No Therapy eTable 3. Health State Utility Values Used in The Model eTable 4. Costs of Therapy eTable 5. Costs Inputs for Outcomes of Therapy eTable 6. Scenario Analysis eFigure 1. Network Map eFigure 2. Population at Risk for Undergoing SOGICE eReferences.
