# Supplementary material for: Humanistic and Economic Burden of Conversion Therapy Among LGBTQ Youths in the United States
Source: JAMA Pediatr. 2022 Mar 7;176(5):493–501. doi: 10.1001/jamapediatrics.2022.0042 (PMC8902682; doi:10.1001/jamapediatrics.2022.0042)
Supplement: Supplement. — eMethods. eTable 1. PICOS Criteria for the SLR eTable 2. Estimates of the Relative Risk of Adverse Health Outcomes: Affirmative Therapy vs No Therapy eTable 3. Health State Utility Values Used in The Model eTable 4. Costs of Therapy eTable 5. Costs Inputs for Outcomes of Therapy eTable 6. Scenario Analysis eFigure 1. Network Map eFigure 2. Population at Risk for Undergoing SOGICE eReferences. [file jamapediatr-e220042-s001.pdf]

Supplementary Online Content

Forsythe A, Pick C, Tremblay G, Malaviya S, Green A, Sandman K. Humanistic and economic burden of conversion therapy among LGBTQ youths in the United States. *JAMA Pediatr.* Published online March 7, 2022. doi:10.1001/jamapediatrics.2022.0042

eMethods.
eTable 1. PICOS Criteria for the SLR
eTable 2. Estimates of the Relative Risk of Adverse Health Outcomes: Affirmative Therapy vs No Therapy
eTable 3. Health State Utility Values Used in The Model
eTable 4. Costs of Therapy
eTable 5. Costs Inputs for Outcomes of Therapy
eTable 6. Scenario Analysis
eFigure 1. Network Map
eFigure 2. Population at Risk for Undergoing SOGICE
eReferences.

This supplementary material has been provided by the authors to give readers additional information about their work.

© 2022 Forsythe A et al. *JAMA Pediatrics*.

eMethods.

Systematic Literature Review Methods and Overall Results

Data Review and Extraction: A systematic literature review (SLR) was conducted in accordance with the Preferred Reporting Items for Systematic Reviews and Meta-Analysis (PRISMA) guidelines.[1,2] The methods followed the principles outlined in the Cochrane Handbook for Systematic Review of Interventions, the Centre for Reviews and Dissemination's Guidance for Undertaking Reviews in Health Care, and the Methods for the Development of the National Institute for Health and Care Excellence's (NICE) Public Health Guidance.[1–4]

Data Sources: Key biomedical literature databases (Medical Literature Analysis and Retrieval System Online [MEDLINE®] and Excerpta Medica Database [Embase®]) were searched via the Ovid platform (database inception to December 2020). MEDLINE® Epub Ahead of Print, In-Process & Other Non-Indexed Citations were searched to ensure that non-indexed citations were retrieved. Bibliographies of relevant review publications were searched to retrieve any additional literature that may have been missed from the Ovid search.

Search Strategy and Inclusion/Exclusion Criteria: The scope of the SLR was defined by Population, Intervention, Comparators, Outcomes, and Study Design (PICOS) criteria (**eTable 1**). The search strategy was constructed to address the following research questions:

- How many individuals, particularly adolescents and young adults, in the US are impacted by SOGICE?
- What are the types and duration of therapy?
- What are the humanistic and economic harms?
- What are the health care resources and costs associated with SOGICE?

The main search terms for SOGICE included "conversion therapy", "reparative therapy", "change therapy", and "gender identity" or "gender role" or "sexual identity" or "transgender" or "sexual minority". The search was not limited by study designs. Only English language studies were included. Among 197 records screened, 28 studies, covering 75,075 subjects and published between 1969 and 2020, met PICOS criteria.

© 2022 Forsythe A et al. *JAMA Pediatrics*.

eTable 1. PICOS Criteria for the SLR

Category	Inclusion Criteria	Exclusion Criteria
Patient population	Individuals who experienced SOGICE*	Individuals who did not experience SOGICE, unless these are used as a control arm
Intervention and comparators	Any forms of SOGICE	Studies not mentioning SOGICE
Outcomes measures	Epidemiology rate of SOGICE	Studies did not have at least one of the outcomes in inclusion criteria
	Patient characteristics: Sub-populations, age at start of therapy	
	Treatment patterns: Types and duration of SOGICE	
	Adverse effects of SOGICE	
	Suicidal attempts	
	Costs of SOGICE	
	Costs of adverse effects of SOGICE	
Study design	Interventional studies	Reviews**
	Real-world evidence studies including:<br>▪ Prospective observational studies<br>▪ Retrospective studies<br>▪ Database analyses	Editorials**<br>Notes/comments/letters
	Cross-sectional survey studies	
	Cost analysis studies and resource use studies	
	Case series/case reports	
Restrictions	English language	Non-English language studies
	No year limitations	

Abbreviations: PICOS, Population, Intervention, Comparators, Outcomes, and Study Design; SLR, systematic literature; SOGICE, sexual orientation and/or gender identity change efforts.
*Inclusion criteria were not restricted by age, but publications focused on adolescents and young adults were preferred as sources of evidence for economic model inputs.
** Relevant reviews were used for bibliographic cross-checking.

© 2022 Forsythe A et al. *JAMA Pediatrics*.

eTable 2. Estimates of the Relative Risk of Adverse Health Outcomes: Affirmative Therapy vs No Therapy

Adverse health outcomes	Relative risk reduction in sourced study	Relative risk in sourced study	Estimated likelihood of health outcomes for affirmative therapy vs no therapy	Low 95% CI	High 95% CI
Anxiety or severe psychological distress[5]	-40%	59.57%	20%	11%	27%
Depression[5]	-34%	65.97%	18%	2%	22%
Alcohol use disorder[5]	-28%	71.83%	30%	24%	35%
Suicidality[6]	-87%	13.49%	3%	0%	6%
Illicit drug abuse[7]	-47%	52.88%	26%	22%	43%

Abbreviation: CI, confidence interval.

An indirect treatment comparison was conducted to estimate the relative risk of adverse health outcomes compared to the estimates obtained for no therapy. The relative risk reduction and relative risk was calculated for each adverse health outcomes from published sources, and then applied to the likelihood estimates obtained for the no therapy intervention.

© 2022 Forsythe A et al. *JAMA Pediatrics*.

eTable 3. Health State Utility Values Used in The Model

Health state	Utility value
Baseline health for LGBTQ youth	0.865[8]
Anxiety/severe psychological distress	0.62[9]
Depression	0.55[10,11]
Alcohol use disorder	0.71[12]
Illicit drug use	0.59[13]
Suicidal attempt	0.26[11]
Fatal suicide/death	0.00

Abbreviation: LGBTQ, lesbian, gay, bisexual, transgender, queer, or questioning.

© 2022 Forsythe A et al. *JAMA Pediatrics*.

eTable 4. Costs of Therapy

Therapy type	Cost per session (US$)	Source
Psychotherapy	142	CPT 90837, American Psychological Association Services Inc.
Religion-based	126	Expert opinion, The Trevor Project, Data on file, 2021

Abbreviation: CPT, current procedural treatment code.

© 2022 Forsythe A et al. *JAMA Pediatrics*.

eTable 5. Costs Inputs for Outcomes of Therapy

Item	Inflation factor	Average cost per patient [US$], (SD/SE/95%CI/range)
Anxiety		
Medical costs (total)	1.23	**2,003 (SE 295)**[14]
Inpatient	1.23	701 (SE 218)[14]
Outpatient	1.23	52 (SE 68)[14]
Emergency department	1.23	46 (SE 23)[14]
Other medical expenses	1.23	100 (SE 48)[14]
Office-based visits	1.23	447 (SE 98)[14]
Prescription medications	1.23	656 (SE 79)[14]
Productivity loss	2.01	2,748 (95% CI 1,426-4,070)[15]
Depression		
Medical costs (total)	1.35	**14,021 (SD 27,626)**[16]
Inpatient	1.35	3,114 (SD 18,825)[16]
Outpatient	1.35	5,889 (SD 13,252)[16]
Emergency department	1.35	561 (SD 2,179)[16]
Other medical expenses	1.35	708 (SD 4,993)[16]
Prescription medications	1.35	3,750 (SD 6,734)[16]
Productivity loss	1.35	5,518 (SD 9,990)[16]
Suicidal Attempts		
Hospitalization		
Medical costs	1.35	13,628[17]
Productivity	1.35	30,244[17]
Emergency Department treated and released		
Medical costs	1.35	4,474[17]
Productivity loss	1.35	1,146[17]
Total		
Medical costs (calculated)		**18,102**
Productivity (calculated)		**31,389**
Fatal suicides		
Medical costs	1.35	6,502[17]
Productivity loss (lifetime)	1.35	2,481,189[17]
Alcohol use disorder		
Medical costs	1.23	1,385 (95% CI 1,199-1,569)[18]

© 2022 Forsythe A et al. *JAMA Pediatrics*.

Item	Inflation factor	Average cost per patient [US$], (SD/SE/95%CI/range)
Rehabilitation		21,000 (range 15,000-27,000)[19]
Productivity	1.56	3,716[20,21]
Substance abuse		
Medical costs	1.23	4,049 (95% CI 2,828-4,042)[18]
Rehabilitation	1.31	21,000 (range 15,000-27,000)[19]
Productivity		1,474[20,22]

Bold font indicates total costs
Abbreviations: CI, confidence interval; SD, standard deviation; SE: standard error.

© 2022 Forsythe A et al. *JAMA Pediatrics*.

eTable 6. Scenario Analysis

Scenario	Discounted Costs ($)			Discounted QALYs			ICURs ($/QALYs)		
	No therapy	SOGICE	Affirmative therapy	No therapy	SOGICE	Affirmative therapy	SOGICE versus no therapy	Affirmative therapy versus no therapy	SOGICE versus Affirmative therapy
Base case*	108,174	206,159	67,844	24.91	23.30	25.84	(61,037)	(43,399)	(54,570)
Utilization of each therapy type based on Blosnich et al data[23]	107,922	281,415	67,781	24.91	21.92	25.84	(58,026)	(43,197)	(54,510)
Exclusion of indirect costs	85,292	148,098	62,056	24.91	23.30	25.84	(39,123)	(25,005)	(33,947)
Re-weighted utility values	85,292	148,098	62,056	24.53	22.73	25.62	(35,032)	(21,315)	(29,845)
Inclusion of fatality due to suicide re-attempt**	112,483	218,461	68,426	24.80	23.00	25.82	(58,675)	(43,110)	(53,051)
Exclusion of fatal suicide attempt	99,569	181,598	66,684	25.10	24.60	25.87	(163,788)	(43,131)	(90,965)

Abbreviations: ICURs, incremental cost-utility ratios; SOGICE, sexual orientation and/or gender identity change efforts; QALYs, quality-adjusted life-years.
*Base case includes the costs and outcomes of a fatal index suicide attempt within the episode period. The total costs include the indirect costs.
**This scenario includes the costs and fatality due to suicide re-attempt (2.3%) over a lifetime after the episode period. It is assumed the re-attempt will occur within 1 year after the episode period.

© 2022 Forsythe A et al. *JAMA Pediatrics*.

eFigure 1. Network Map

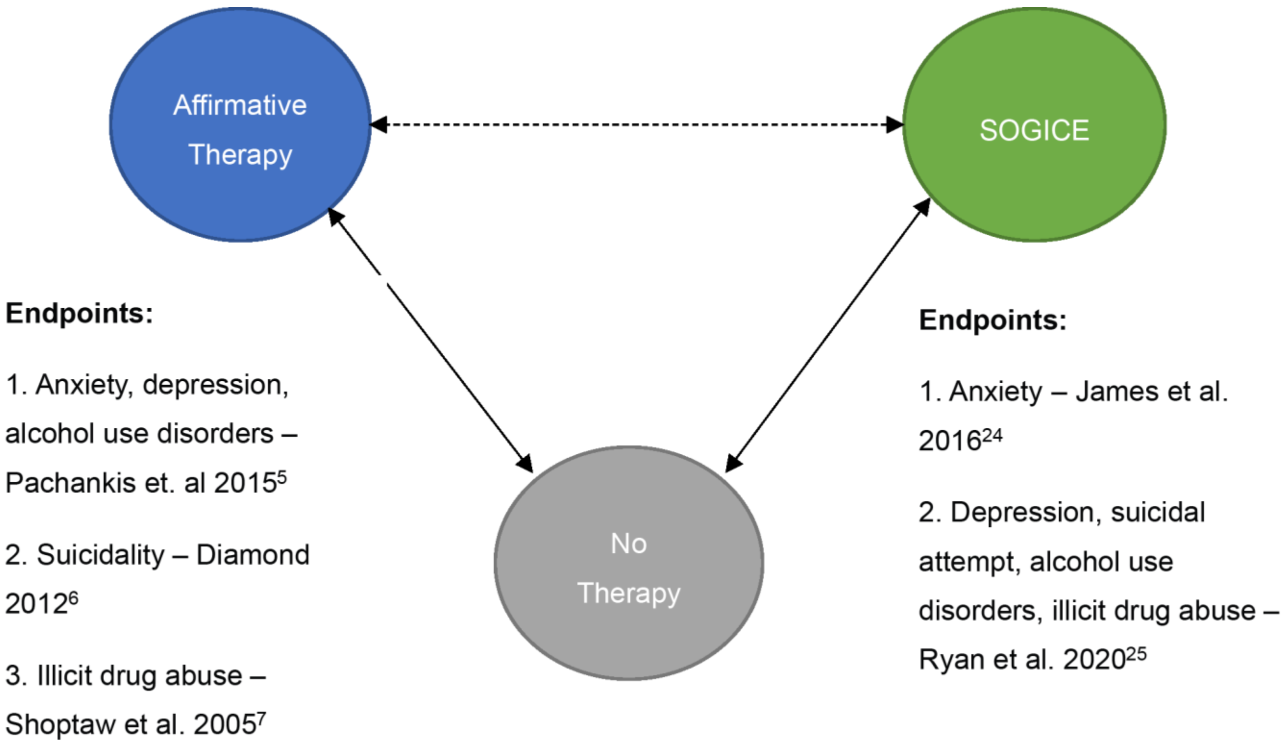

Abbreviation: SOGICE, sexual orientation and/or gender identity change efforts.

© 2022 Forsythe A et al. *JAMA Pediatrics*.

eFigure 2. Population at Risk for Undergoing SOGICE

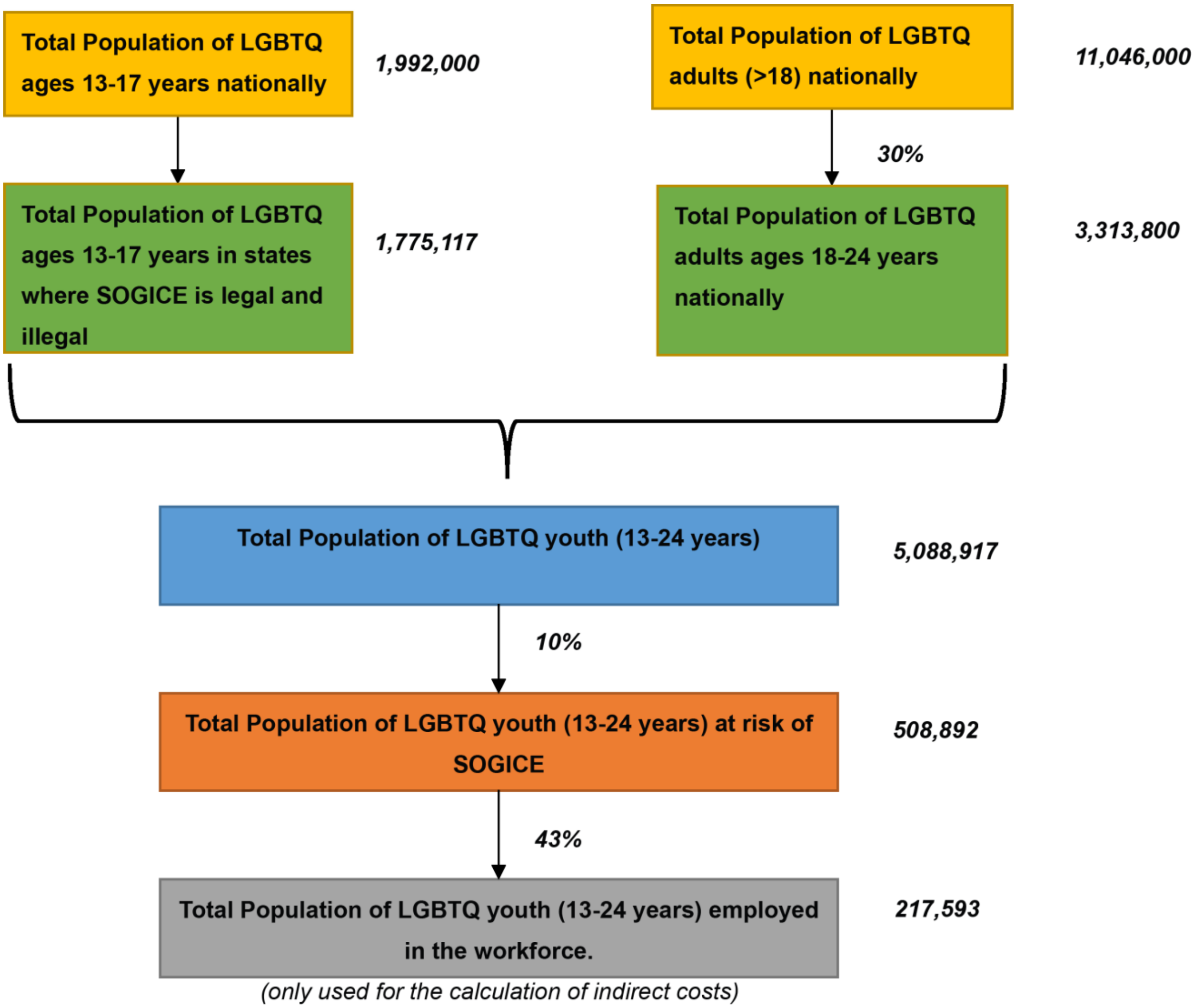

Abbreviations: LGBTQ, lesbian, gay, bisexual, transgender, queer, or questioning; SOGICE, sexual orientation and/or gender identity change efforts. Note: at the time of the analysis, SOGICE was banned in 20 states.

© 2022 Forsythe A et al. *JAMA Pediatrics*.

eReferences.

1. Moher D, Liberati A, Tetzlaff J, Altman DG, PRISMA Group. Preferred reporting items for systematic reviews and meta-analyses: the PRISMA statement. *BMJ*. 2009;339:b2535-b2535. doi:10.1136/bmj.b2535

2. NICE. Methods for the development of NICE public health guidance (third edition). In: ; 2012. Accessed December 23, 2019. https://www.ncbi.nlm.nih.gov/books/NBK395862/pdf/Bookshelf_NBK395862.pdf

3. Centre for Reviews and Dissemination. Systematic reviews: CRD's guidance for undertaking reviews in health care. In: ; 2009. Accessed December 23, 2019. https://www.york.ac.uk/media/crd/Systematic_Reviews.pdf

4. Higgins J, Thomas J, Chandler J, Cumpston M. Cochrane Handbook for Systematic Reviews of Interventions | Cochrane Training. In: 6.0. John Wiley & Sons; 2019. Accessed December 23, 2019. https://training.cochrane.org/handbook/current

5. Pachankis JE, Hatzenbuehler ML, Rendina HJ, Safren SA, Parsons JT. LGB-affirmative cognitive-behavioral therapy for young adult gay and bisexual men: A randomized controlled trial of a transdiagnostic minority stress approach. *J Consult Clin Psychol*. 2015;83(5):875-889. doi:10.1037/ccp0000037

6. Diamond GM, Diamond GS, Levy S, Closs C, Ladipo T, Siqueland L. Attachment-based family therapy for suicidal lesbian, gay, and bisexual adolescents: a treatment development study and open trial with preliminary findings. *Psychotherapy (Chic)*. 2012;49(1):62-71. doi:10.1037/a0026247

7. Shoptaw S, Reback CJ, Peck JA, et al. Behavioral treatment approaches for methamphetamine dependence and HIV-related sexual risk behaviors among urban gay and bisexual men. *Drug Alcohol Depend*. 2005;78(2):125-134. doi:10.1016/j.drugalcdep.2004.10.004

8. Padula WV, Heru S, Campbell JD. Societal Implications of Health Insurance Coverage for Medically Necessary Services in the U.S. Transgender Population: A Cost-Effectiveness Analysis. *J Gen Intern Med*. 2016;31(4):394-401. doi:10.1007/s11606-015-3529-6

9. Chatterton ML, Rapee RM, Catchpool M, et al. Economic evaluation of stepped care for the management of childhood anxiety disorders: Results from a randomised trial. *Aust N Z J Psychiatry*. 2019;53(7):673-682. doi:10.1177/0004867418823272

10. Sobocki P, Ekman M, Agren H, et al. Health-related quality of life measured with EQ-5D in patients treated for depression in primary care. *Value Health*. 2007;10(2):153-160. doi:10.1111/j.1524-4733.2006.00162.x

11. Mrazek DA, Hornberger JC, Altar CA, Degtiar I. A review of the clinical, economic, and societal burden of treatment-resistant depression: 1996-2013. *Psychiatr Serv*. 2014;65(8):977-987. doi:10.1176/appi.ps.201300059

12. Sonntag M, König H-H, Konnopka A. The responsiveness of the EQ-5D and time trade-off scores in schizophrenia, affective disorders, and alcohol addiction. *Health Qual Life Outcomes*. 2015;13:114. doi:10.1186/s12955-015-0315-4

13. Rand K, Arnevik EA, Walderhaug E. Quality of life among patients seeking treatment for substance use disorder, as measured with the EQ-5D-3L. *Journal of Patient-Reported Outcomes*. 2020;4(1):92. doi:10.1186/s41687-020-00247-0

14. Shirneshan E, Bailey J, Relyea G, Franklin BE, Solomon DK, Brown LM. Incremental direct medical expenditures associated with anxiety disorders for the U.S. adult population: evidence from the Medical Expenditure Panel Survey. *J Anxiety Disord*. 2013;27(7):720-727. doi:10.1016/j.janxdis.2013.09.009

© 2022 Forsythe A et al. *JAMA Pediatrics*.

15. Marciniak M, Lage MJ, Landbloom RP, Dunayevich E, Bowman L. Medical and productivity costs of anxiety disorders: Case control study. *Depress Anxiety*. 2004;19(2):112-120. doi:10.1002/da.10131

16. Greenberg PE, Fournier A-A, Sisitsky T, Pike CT, Kessler and RC. The Economic Burden of Adults With Major Depressive Disorder in the United States (2005 and 2010). 2015;76. doi:10.4088/JCP.14m09298

17. Centers for Disease Control. Cost of Injury & Calculators | WISQARS | Injury Center | CDC. Published June 30, 2020. Accessed March 1, 2021. https://www.cdc.gov/injury/wisqars/cost/index.html

18. Gryczynski J, Schwartz RP, O'Grady KE, Restivo L, Mitchell SG, Jaffe JH. Understanding Patterns Of High-Cost Health Care Use Across Different Substance User Groups. *Health Aff (Millwood)*. 2016;35(1):12-19. doi:10.1377/hlthaff.2015.0618

19. American Addiction Centers, 2020. How Much Does Alcohol Rehab Cost? Residential & Outpatient Care Costs. American Addiction Centers. Accessed March 1, 2021. https://americanaddictioncenters.org/alcohol-rehab/cost

20. US Bureau of Labor Statistics. Usual Weekly Earnings Summary. Published December 2020. Accessed March 1, 2021. https://www.bls.gov/news.release/wkyeng.nr0.htm

21. Bouchery EE, Harwood HJ, Sacks JJ, Simon CJ, Brewer RD. Economic Costs of Excessive Alcohol Consumption in the U.S., 2006. *American Journal of Preventive Medicine*. 2011;41(5):516-524. doi:10.1016/j.amepre.2011.06.045

22. Foster WH, Vaughan RD. Absenteeism and business costs: Does substance abuse matter? *Journal of Substance Abuse Treatment*. 2005;28(1):27-33. doi:10.1016/j.jsat.2004.10.003

23. Blosnich JR, Henderson ER, Coulter RWS, Goldbach JT, Meyer IH. Sexual Orientation Change Efforts, Adverse Childhood Experiences, and Suicide Ideation and Attempt Among Sexual Minority Adults, United States, 2016–2018. *Am J Public Health*. 2020;110(7):1024-1030. doi:10.2105/AJPH.2020.305637

24. James S, Herman J, Rankin S, Keisling M, Mottet L, Anafi M. The Report of the 2015 U.S. Transgender Survey. Washington, DC: National Center for Transgender Equality, 2016. https://ncvc.dspacedirect.org/handle/20.500.11990/1299.

25. Ryan C, Toomey RB, Diaz RM, Russell ST. Parent-Initiated Sexual Orientation Change Efforts With LGBT Adolescents: Implications for Young Adult Mental Health and Adjustment. *Journal of Homosexuality.* 2020;67(2):159-73. https://doi.org/10.1080/00918369.2018.1538407.

© 2022 Forsythe A et al. *JAMA Pediatrics*.
